# Regulatory Effects of Pumpkin Seed Extract on Glucose Metabolism and Insulin Signaling in Diabetic Models

**DOI:** 10.1021/acsomega.5c13268

**Published:** 2026-03-27

**Authors:** Yu-Cheng Lin, Pang-Kuei Hsu, Min-Hsiung Pan

**Affiliations:** † Institute of Food Science and Technology, 33561 National Taiwan University, Taipei 10617, Taiwan; ‡ Greenyn Biotechnology Co., Ltd., Daya, Taichung City 428015, Taiwan; § Department of Medical Research, China Medical University Hospital, China Medical University, Taichung City 40402, Taiwan; ∥ Department of Health and Nutrition Biotechnology, Asia University, Taichung City 41354, Taiwan

## Abstract

Diabetes mellitus is a chronic metabolic disorder characterized by impaired insulin signaling and glucose homeostasis. In this study, a defatted pumpkin seed extract (PSE), prepared via supercritical fluid extraction, was investigated for its potential antidiabetic effects. In streptozotocin-induced diabetic rats, PSE supplementation significantly reduced fasting blood glucose, HbA1c (glycated hemoglobin), and fasting insulin levels and homeostasis model assessment of insulin resistance. Oral glucose tolerance tests revealed enhanced insulin sensitivity in a dose-dependent manner. RNA sequencing of insulin-resistant C2C12 myotubes revealed differentially expressed genes related to PI3K/Akt signaling and mitochondrial function. Western blot analysis further demonstrated increased phosphorylation of the insulin receptor and Akt, suggesting the PSE-mediated activation of insulin signaling in skeletal muscle. These preclinical findings warrant further clinical and safety evaluation of PSE and PSE-02 as nutraceutical candidates.

## Graphical abstract

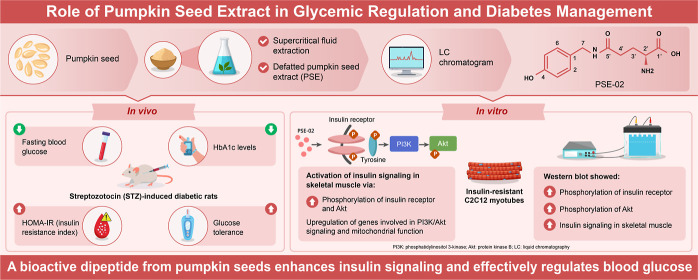

## Introduction

Diabetes mellitus (DM) is a serious noncommunicable disease and one of the main causes of death worldwide.^1^ According to the World Health Organization, DM accounted for approximately 1.5 million deaths in 2019, and the number of people with DM worldwide is projected to increase from 537 million in 2021 to 643 million by 2030.^1,2^ DM also increases the risk of several diseases (e.g., cardiovascular disease, kidney damage, and vision loss) and incurs a heavy healthcare burden globally.^3^ For example, the annual health expenditure for diabetes in the USA is approximately USD 294.6 billion.^4^ Type II DM (T2DM) accounts for more than 90% of DM cases, and it results from a lack of insulin production by pancreatic β-cells and insulin resistance.^5^ Medication and lifestyle changes are conventionally applied to individuals with DM for effective glycemic control; however, some synthetic diabetes drugs may induce undesirable side effects, such as diarrhea and nausea.^6,7^ Hence, complementary and alternative medicine has recently become a promising approach for diabetes management, especially herbal remedies that have hypoglycemic effects.^7^ Among them, pumpkin is known for its potential for glycemic control.^8^

Pumpkin belongs to the family *Cucurbitaceae*, which includes 130 genera and 800 species.^9^ Pumpkin seeds mainly consist of protein (14.3–38.0%) and fat (21.9–54.9%) and contain an array of functional components, such as tocopherols, carotenoids, unsaturated fatty acids, phytosterols, triterpenoids, and phytophenols.^10,11^ Pharmacological studies revealed that pumpkin seed extracts (PSEs) possess hepatoprotective, antibacterial, antioxidant, hypocholesterolemic, hypotensive, anticancer, and antidiabetic activities.^11,12^ Tocopherol extracted from pumpkin seeds remarkably improved plasma glucose and insulin levels in diabetic rats.^13^ An aqueous extract of pumpkin seeds reduced fasting blood glucose (FBG) levels in mild and severely diabetic Wistar rats by 26.15% and 39.33%, respectively, after a 28 day treatment.^14^ Moreover, Abdelkader et al.^14^ demonstrated that pumpkin seed protein ameliorated plasma glucose and insulin levels and improved glucose tolerance in rats treated with or without fructose.^15^ Nonetheless, the mechanism underlying the hypoglycemic effect of PSEs or the antidiabetic peptides in PSEs is unclear.

This study aimed to determine the potential role of PSE in glucose regulation and to elucidate the mechanisms underlying its hypoglycemic effects using streptozotocin (STZ)-induced diabetic rats. Additionally, cell-based RNA sequencing (RNA-seq) analysis was conducted to identify differentially expressed genes (DEGs) and metabolic pathways influenced by PSE treatment, providing insights into its molecular actions. This comprehensive approach not only helps to validate the potential of defatted pumpkin seeds as a functional dietary ingredient for diabetes management but also lays the groundwork for future investigations into their bioactive components and therapeutic applications.

## Methods

### Materials and Reagents

Supercritical fluid defatted pumpkin seed residues (obtained after SF-CO_2_ extraction) were obtained from Greenyn Biotechnology Co., Ltd. (Taichung, Taiwan). A BCA assay kit was purchased from Beyotime (Nanjing, China). All other chemicals and biochemical kits were purchased from Sigma-Aldrich (St. Louis, MO, USA).

### Preparation of PSE

A 40 g sample of pumpkin seed residues was weighted and ultrasonication-extracted with 800 mL solvent (H_2_O containing 5% acetonitrile) at 8 °C ± 2 °C. The extracts were centrifugally separated at 9000 rpm for 8 min, and the supernatant was collected and lyophilized for further use.

The obtained extract was separated by medium-pressure liquid chromatography (MPLC) with LiChroprep RP-18 to afford seven fractions (Fr. 1–Fr. 7) by elution with a linear gradient of a mixture of MeOH and H_2_O (10:90–90:10). Fr. 1 was subjected to preparative high-performance liquid chromatogram (HPLC) with a Thermo ODS Hypersil column (10 × 250 mm, 5 μm) and eluted by a mixture of MeOH and H_2_O (10:90) to yield compound PSE-05. Fr. 2 was purified by preparative HPLC using an isocratic solvent system of MeOH–H_2_O (30:70) to obtain compound PSE-04. Fr. 3 was loaded onto a preparative HPLC system and eluted with a mixture of MeOH and H_2_O (20:80) to yield compounds PSE-03 and PSE-02. Fr. 4 was subjected to Sephadex LH-20 chromatography and eluted with MeOH to yield compound PSE-07. Fr. 5 was applied to an RP-18 column by MPLC, eluted with a gradient solvent of MeOH and H_2_O (from 10:90 to 90:10), and further purified by HPLC with a mixture of MeOH–H_2_O (35:65) to obtain compounds PSE-01 and PSE-06. The structures of all of the isolates were determined by NMR analysis (structural characterization only). PSE-02 was purified and selected for downstream functional assays to evaluate its biological activity.

### HPLC and NMR Analysis of PSE

The PSE was analyzed by using a Shimadzu LC-2030C HPLC system equipped with a diode array UV–vis detector. The injection volume was 10 μL, and the flow rate was 1 mL/min. Mobile phase A comprised MeOH with 0.1% formic acid, while mobile phase B consisted of H_2_O containing 0.1% formic acid. The gradient system was as follows: 5% mobile phase A from 0 to 5 min, 5–20% mobile phase A from 5 to 20 min, 20–100% mobile phase A from 20 to 45 min; 100% mobile phase A from 45 to 50 min, 5% mobile phase A from 50 to 50.1 min; it was held for 60 min. The separated chemicals were quantified by measuring their absorbances at 220 nm. The column oven was set to 40 °C.

^1^H NMR and ^13^C NMR spectra were recorded on a Varian Unity Plus 400 MHz spectrometer by using TMS as an internal standard. Chemical shifts were reported in parts per million (ppm), and the coupling constants (J) were expressed in Hz. ESI-MS measurements were performed using an Agilent 6420 Triple Quadrupole LC–MS spectrometer equipped with an ESI ionization source. MPLC was performed on a Combi*Flash* Rf^+^ instrument (Teledyne ISCO, Lincoln, USA). Further purification of some compounds was achieved by a preparative HPLC (Shimadzu LC-10AT) system equipped with a Thermo ODS Hypersil column (10 × 250 mm, 5 μm). TLC was performed on silica gel 60, F254 (0.20 nm, Merck), and spots were viewed under ultraviolet light at 254 and 365 nm and stained by spraying with 50% H_2_SO_4_ followed by heating on a hot plate. For column chromatography, LiChroprep RP-18 (Merck KGaA) and Sephadex LH-20 (Pharmacia Fine Chemicals AB, Uppsala, Sweden) were used. Methanol (MeOH, HPLC grade) and formic acid (analytical grade) were purchased from Sigma Chemical Co. (St. Louis, MO, USA). The purity of PSE-02 was evaluated by analytical HPLC (UV detection), and its identity was confirmed by LC–ESI–MS.

### Cell-Based Functional Assays, RNA-seq, and qPCR

The mouse myoblast cell line C2C12 was obtained from the Bioresources Collection & Research Center (BCRC, FIRDI, Hsinchu, Taiwan). Cells were maintained in Dulbecco’s modified Eagle’s medium (DMEM) supplemented with 10% (v/v) fetal bovine serum, streptomycin (100 U/mL), and penicillin (100 U/mL) at 37 °C in a humidified incubator with 5% CO_2_. When the cells reached 70–80% confluence, differentiation was induced by switching to DMEM containing 2% horse serum for 3 days to form mature myotubes.

To establish an insulin resistance model,^16^ differentiated C2C12 myotubes were cultured in a serum-free medium composed of an equal mixture of MCDB 201 and Ham’s F-12 (1:1, v/v), either in the absence (MF) or continuous presence (MFI) of insulin (100 nM) for 72 h (3 days). During the induction period, the culture medium (±insulin) was refreshed every 24 h with freshly prepared insulin-containing medium to maintain stable treatment conditions. For downstream assays, myotubes were treated with PSE-02 at the indicated concentrations for the last 24 h of the induction period. As a positive control for insulin responsiveness, MF myotubes were subjected to acute insulin stimulation (10 nM, 15 min) immediately prior to harvest. Following treatment, total cellular proteins were extracted for downstream analyses, and RNA-seq analysis was outsourced to Tools Biotechnology Co., Ltd. (Taiwan). For quantitative real-time PCR (qPCR) validation, cells were harvested and washed twice with ice-cold phosphate-buffered saline, and total RNA was extracted using the FastPure Cell/Tissue Total RNA Isolation Kit V2 (Vazyme, Nanjing, China) according to the manufacturer’s instructions. RNA purity and concentration were assessed by using an Epoch 2 microplate spectrophotometer (BioTek, Winooski, VT, USA). First-strand cDNA was synthesized from 1 μg of total RNA using a HiScript III All-in-one RT SuperMix kit (Vazyme). qPCR was performed with a 2× MorreSYBR qPCR Master Mix (MORREBIO, Taipei, Taiwan) on a CFX Duet Real-Time PCR System (Bio-Rad, Hercules, CA, USA). Relative mRNA expression levels were calculated using the 2^–ΔΔ*Ct*^ method, with glyceraldehyde 3-phosphate dehydrogenase (GAPDH) serving as the internal control. For cell-based assays, experiments were performed in three independent biological replicates (*n* = 3).

### Induction of T2DM by High-Fat Diet Feeding and Low-Dose STZ Treatment

Forty male rats (8 weeks old) were purchased from BioLASCO Taiwan Co. Ltd. The rats were kept in a temperature-controlled environment (25 ± 5 °C) with a relative humidity of 55 ± 10% and a 12 h light–dark cycle. A commercial chow diet and water were provided ad libitum. After 1 week of acclimatization, the rats were randomly assigned to receive either a normal chow diet or a high-fat diet (HFD), and T2DM was induced in the HFD-fed animals by low-dose STZ, a widely used protocol to model type 2 diabetes characterized by both diet-induced insulin resistance and partial β-cell dysfunction.

On a caloric basis, the HFD consisted of fuel energy of 5.1 kcal/g, comprising 60.3% calories from fat, 18.3% from protein, and 21.4% from carbohydrate (TD.06414, ENVIGO, USA). Following acclimatization and HFD feeding, rats were intraperitoneally injected with a low dose of STZ [25 mg/kg dissolved in ice-cold 0.01 M citrate buffer (pH 4.4)], as described by Srinivasan et al.^17^ Rats with diabetes (blood glucose level >220 mg/dL) were used for the experiments. No mortality was observed during induction. Diabetic rats were fed an HFD continuously during the study. Rats were randomly divided into five experimental groups: (1) normal control group (CON): chow diet, oral gavage with 1 mL of saline; (2) DM group: HFD + STZ-induced T2DM rats were orally administered 1 mL of saline for 8 weeks; (3) LD group: DM rats were treated with 25.0 mg/kg PSE via oral gavage for 8 weeks; (4) HD group: DM rats were treated with 50.0 mg/kg PSE via oral gavage for 8 weeks; and (5) PSE-02-treated group (PSE-02): DM rats received 0.06 mg/kg purified PSE-02 via oral gavage daily for 8 weeks (*n* = 8 rats per group).

The PSE doses (25.0 and 50.0 mg/kg) were selected to represent low and moderate intake levels of a concentrated-food-derived ingredient and fall within the range commonly used in rodent studies of plant protein extracts. Using body surface area-based conversion (Km_rat = 6, Km_human = 37), these doses correspond to human equivalent doses of approximately 4.1 and 8.1 mg/kg (i.e., ∼240 and 480 mg/day for a 60 kg adult), whereas the PSE-02 dose (0.06 mg/kg) corresponds to approximately 0.01 mg/kg (∼0.6 mg/day) in humans. Throughout the 8-week intervention, no treatment-related mortality or overt signs of distress were observed, suggesting that the selected doses were well tolerated under the experimental conditions.

### Serum Biochemical Analysis

Blood glucose levels were determined for 18 h fasted rats (access to water ad libitum) using blood samples obtained from the tails using a glucometer (Model GB, Roche, Mannheim, Germany).^18^ Briefly, blood was centrifuged at 800*g* for 30 min at 4 °C to obtain the serum and then frozen at −80 °C. Serum insulin levels were quantified using ELISA kits (Abbkine, China). Homeostasis model assessment of insulin resistance (HOMA-IR) was calculated as fasting insulin (μU/mL) × fasting glucose (mmol/L)/22.5. Fasting glucose values measured in mg/dL were converted to mmol/L using mmol/L = mg/dL ÷ 18 (equivalent to multiplying by 0.0555). This is mathematically equivalent to HOMA-IR = fasting insulin (μU/mL) × fasting glucose (mg/dL)/405.^19^ The oral glucose tolerance test (OGTT) was performed in the ninth week (*n* = 8 rats per group). After a 12 h overnight fast and baseline sampling, a 2 g/kg glucose solution was administered orally by gavage, followed by collection of blood samples from the tail at 30, 60, 90, and 120 min for blood glucose determination. Area under the curve (AUC) determined by glucose levels at baseline and 120 min after glucose overload was used to calculate the AUC-OGTT (*n* = 8 rats per group).

### Western Blotting

Tissue extraction and Western blotting were performed as previously described.^20^ Skeletal muscle was homogenized using liquid nitrogen and ice-cold RIPA buffer (20 mM HEPES, 50 mM Tris–HCl, 150 mM NaCl, 1% Nonidet P-40, and 0.5% sodium deoxycholate, pH 7.4) containing protease and phosphatase inhibitor cocktails. The supernatant was collected after centrifugation at 12,000*g* for 15 min at 4 °C. The protein content was assessed using a BCA assay kit (Beyotime, Nanjing, China). Protein samples were separated via 8% sodium dodecyl sulfate-polyacrylamide gel electrophoresis and then transferred to polyvinylidene difluoride membranes (Millipore, Billerica, MA, USA). The membranes were then incubated with the primary antibody. β-Actin was used as the loading control. Anti-Akt and antiphospho-Akt antibodies were purchased from Cell Signaling Technology. Anti-IR and antiphospho-IR antibodies were purchased from Santa Cruz Biotechnology. Anti-PDK1 and anti-PI3K were obtained from GeneTex. After extensive washing with TBST, the membranes were incubated for 60 min with antirabbit or antimouse HRP-coupled secondary antibodies at a 1:10,000 dilution. Immunoreactive bands were detected by using the MGIS-21-C2-6M imaging system (TOPBIO, Taiwan).

### Statistical Analysis

All experimental data are expressed as mean ± standard deviation (SD). Statistical analyses were performed using the SigmaPlot 14.0 software (Systat Software, Inc., San Jose, CA, USA). Differences between groups were assessed using one-way analysis of variance (ANOVA), followed by Tukey’s post hoc test for multiple comparisons. An unpaired student’s *t*-test was used for comparisons between two groups. For RNA-seq analysis, Kyoto Encyclopedia of Genes and Genomes (KEGG) pathway enrichment analyses were performed using DAVID. Statistical significance was defined as *p* < 0.05.

## Results and Discussion

### Structural Identification and Bioinformatic Analysis of PSE-02

The HPLC of PSE is shown in Figure 1. Seven peaks (PSE-01 to PSE-07) with high signal intensities and good resolutions were selected for isolation and identification of major components, and the results are presented in Figure 2. To comprehensively characterize the chemical profile of the defatted PSE, we purified and structurally elucidated multiple constituents (PSE-01∼PSE-07). However, the present study mechanistically focuses on PSE-02, whereas the bioactivities of the other components will be explored in future work. The ESI-MS spectrum of PSE-02 exhibited a pseudomolecular ion peak at *m*/*z* = 253 [M + H]^+^, corresponding to a molecular weight of 252. In the ^1^H NMR spectrum (Table 1), the aromatic region displayed characteristic A_2_B_2_ splitting patterns with signals at δH 7.10 (2H, dd, *J* = 8.4, 1.6 Hz, H-3,5) and δH 6.76 (2H, dd, *J* = 8.4, 1.6 Hz, H-2,6), indicative of a para-substituted phenyl group. Additionally, the singlet proton signal at δH 4.18 (2H, s) was attributed to the methylene group attached to the para-substituted benzyl moiety. The ^13^C NMR and DEPT spectra revealed 12 carbon signals, including four quaternary carbons, five methine carbons, and three methylene carbons. Further structural elucidation was performed by using ^1^H–^1^H COSY and HMBC spectra (Figure 3). In the COSY spectrum, correlations were observed for the glutamine moiety, including δH 3.64 (1H, t, *J* = 6.2 Hz, H-2′), δH 2.33 (2H, m, H-3′), and δH 2.02 (2H, m, H-4′). The HMBC correlations provided additional structural insights, namely δH 4.18 (2H, s, H-7) correlated with δC 115.4 (C-2,6) and δC 174.3 (C-5′), while δH 2.02 (2H, m, H-3′) correlated with δC 173.9 (C-1′), δC 54.1 (C-2′), and δC 174.3 (C-5′). Given the biosynthetic origin from plant protein, the glutamine residue in PSE-02 was assumed to be in the L-configuration; however, its absolute stereochemistry was not formally determined in the present study and represents a limitation for full structural characterization.

**Figure 1:**
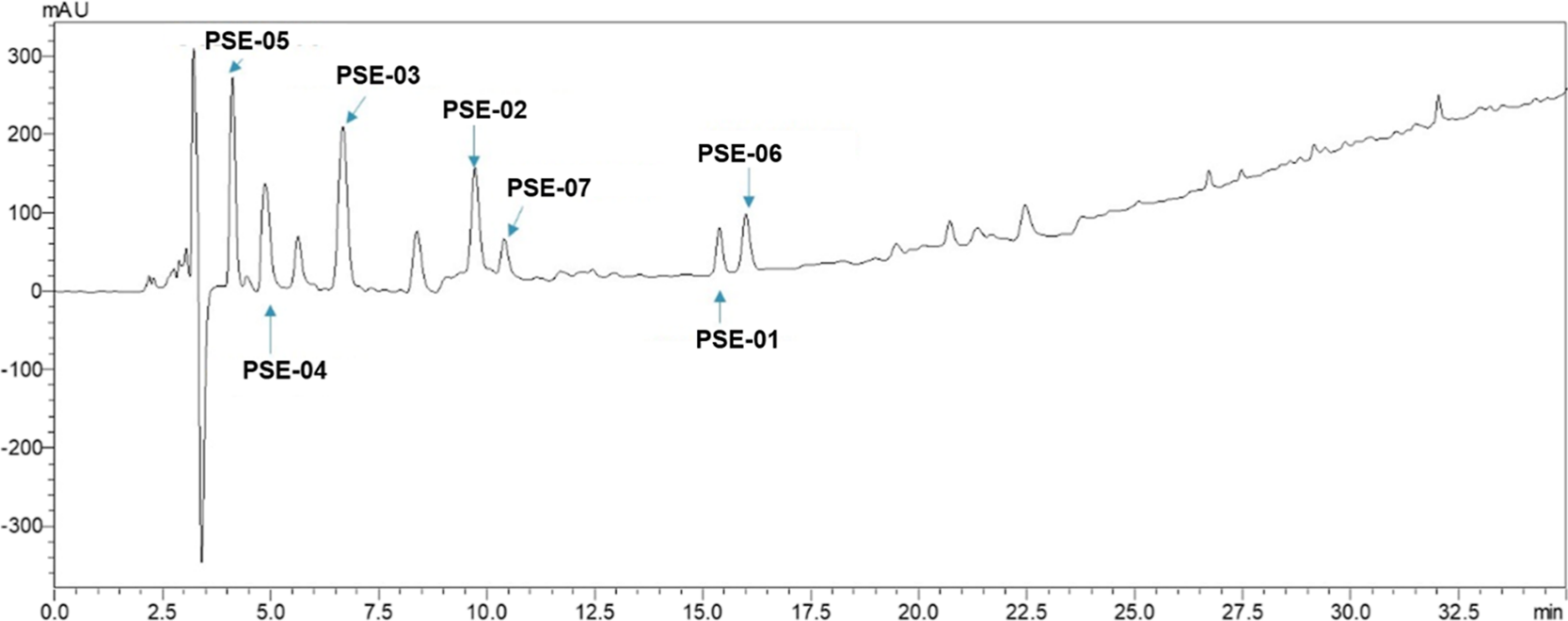
HPLC chromatogram of PSE with retention times corresponding to PSE-01 to PSE-07. *PSE-01∼07: Purified fractions of pumpkin seed extracts 01∼07.

**Figure 2:**
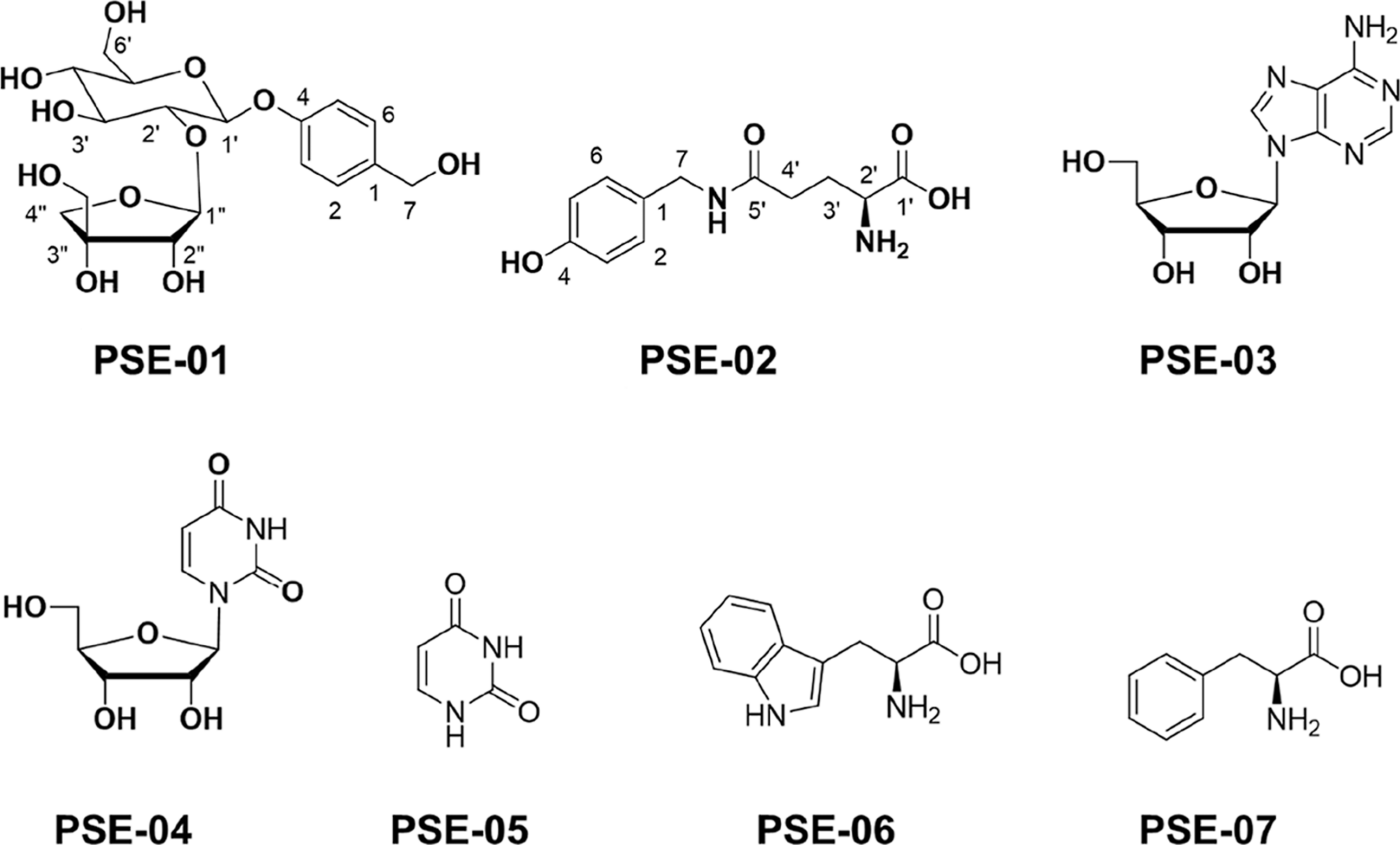
Chemical structures of the major components isolated from PSE-01 to PSE-07 fractions of PSE. *PSE-01∼07: Purified fractions of pumpkin seed extracts 01∼07.

**Table 1:** ^1^H (400 MHz) and ^13^C (100 MHz) NMR Spectroscopic Data of PSE-02^a^

	PSE-02^b^	
position	δ_H_ mult. (*J* in Hz)	δ_c_
1	-	129.8
2	6.76 dd (8.4, 1.6)	115.4
3	7.10 dd (8.4, 1.6)	128.9
4	-	154.6
5	7.10 dd (8.4, 1.6)	128.9
6	6.76 dd (8.4, 1.6)	115.4
7	4.18 s	42.5
1′	-	173.9
2′	3.64 t (6.2)	54.1
3′	2.02 m	26.4
4′	2.33 m	31.5
5′	-	174.3

a The d-solvent was CD_3_OD.

b The d-solvent was D_2_O.

**Figure 3:**
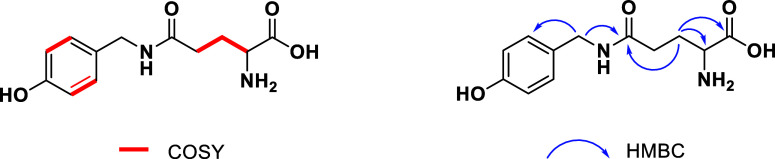
Key COSY and HMBC correlations observed in the NMR analysis of PSE-02. *COSY: Correlation spectroscopy. *HMBC: Heteronuclear multiple bond correlation.

These correlations confirmed that the 4-hydroxybenzyl moiety is linked to the glutamine backbone via an amide bond. Based on the comprehensive spectroscopic data, PSE-02 was identified as a novel dipeptide, structurally characterized as *N* ^5^-(4-hydroxybenzyl)-glutamine.^21^ This unique structure prompted further investigations into its potential role in modulating glucose metabolism at the molecular level.

To elucidate its mechanism of action, RNA-seq was performed on insulin-resistant C2C12 myotubes treated with PSE-02. KEGG pathway enrichment analysis revealed a significant modulation of genes involved in mitochondrial respiration, glycan biosynthesis, and insulin signaling (Table 2). Specifically, Cox8a and Cox7b, encoding cytochrome *c* oxidase subunits essential for oxidative phosphorylation and ATP synthesis, were upregulated (log_2_ FC = 1.677 and 1.433, respectively; *p* < 0.01). This suggests enhanced mitochondrial activity, which is often impaired in the insulin-resistant state.^22^ Notably, previous systems-level analyses have shown that coordinated but modest transcriptional changes in mitochondrial gene sets can still be functionally relevant for oxidative phosphorylation capacity, as demonstrated by Mootha et al. in skeletal muscle.^22^ Consistently, qPCR validation (Table 3) showed a dose-dependent increase of Cox8a and Cox7b expression (approximately 1.1–1.3-fold vs control) in PSE-02-treated myotubes, supporting the transcriptomic indication that PSE-02 improves mitochondrial respiratory function.

**Table 2:** Results of KEGG Analysis of PSE-02^a^ ^,^ ^b^

gene symbol	entrez ID	KEGG pathway	log_2_ fold change	*p*-value
Cox8a	12868	cytochrome *c* oxidase	1.677	0.00045
Cox7b	66142	cytochrome *c* oxidase	1.433	0.0093
C1galt1c1	59048	O-glycan biosynthesis	–1.231	0.0332
Pik3r1	18708	PI3K/AKT signaling pathway	–0.728	0.0398
Akt3	23797	PI3K/AKT signaling pathway	–0.594	0.0448
Smarca5	93762	chromatin remodeling	–0.678	0.0268

a RNA-seq was performed using three independent biological replicates per condition (*n* = 3).

b *KEGG: Kyoto Encyclopedia of Genes and Genomes.

**Table 3:** qPCR Validation of Selected DEGs in PSE-02-Treated Insulin-Resistant C2C12 Myotubes^a^

gene	control	PSE-02 1 nM	PSE-02 5 nM	PSE-02 10 nM
Cox8a	1.000 ± 0.025	0.976 ± 0.051	1.131 ± 0.097	1.324 ± 0.158
Cox7b	1.002 ± 0.063	1.165 ± 0.063	1.203 ± 0.032	1.324 ± 0.290
Akt3	1.002 ± 0.072	0.537 ± 0.178	0.637 ± 0.091	0.615 ± 0.152

a Data are expressed as relative mRNA expression normalized to the control group (fold change, mean ± SD; *n* = 3 independent experiments). Insulin-resistant C2C12 myotubes were treated with PSE-02 (1, 5, or 10 nM) for 24 h prior to RNA extraction and qPCR analysis.

In parallel, C1galt1c1, a gene involved in O-glycan biosynthesis, was significantly downregulated (log_2_ FC = −1.231, *p* = 0.0332), indicating potential changes in protein glycosylation that may affect insulin receptor (IR) function or glucose transporter trafficking. Notably, PSE-02 also altered the expression of Pik3r1 and Akt3, key components of the PI3K/Akt signaling cascade, with negative log_2_ FC values of −0.728 and −0.594, respectively. These results suggest a context-dependent regulation of insulin-signaling intermediates,^23–25^ possibly through feedback modulation or compensatory expression under chronic insulin resistance. In agreement with the RNA-seq data, qPCR analysis confirmed a marked reduction of Akt3 mRNA levels (∼0.5–0.6-fold vs control; Table 3), and a similar work in other cell systems has indicated that even relatively small log_2_ fold changes can exert biologically meaningful effects when they occur within tightly connected regulatory networks.^26^

Additionally, suppression of Smarca5, a gene involved in chromatin remodeling (log_2_ FC = −0.678, *p* = 0.0268), may reflect epigenetic adjustments triggered by PSE-02, which could further influence the transcriptional regulation of metabolic genes.

Collectively, these transcriptomic and qPCR findings demonstrate that PSE-02 exerts pleiotropic effects on pathways critical for energy metabolism and insulin signaling. This is consistent with the broader notion that subtle but coordinated transcriptomic shifts can underpin robust physiological adaptations in metabolic tissues, as emphasized in recent integrative genomic analyses.^27^ This evidence provided a mechanistic foundation for the glycemic improvements observed in subsequent in vivo experiments using PSE-derived compounds.

Collectively, these transcriptomic findings demonstrate that PSE-02 exerts pleiotropic effects on pathways critical for energy metabolism and insulin signaling. This evidence provided a mechanistic foundation for the glycemic improvements observed in subsequent in vivo experiments using PSE-derived compounds.

### PSE Improves Glycemic Control and Glucose Tolerance in Diabetic Rats: Comparative Insights with PSE-02

Sustainable extraction techniques, such as supercritical carbon dioxide (SF–CO_2_) extraction, have been widely adopted to isolate bioactive compounds while preserving their structural and functional integrity.^28^ In this study, SF-CO_2_ was employed to efficiently remove lipids from pumpkin seeds, resulting in a protein-enriched extract (PSE). This defatting process enabled us to specifically examine the biological effects attributable to the protein fraction, excluding potential confounding influences of lipid components, which are also known to exhibit hypoglycemic activity.^7^ Despite increasing evidence supporting the antidiabetic potential of pumpkin seed proteins, the specific bioactive constituents responsible for these effects have not been clearly identified. To address this gap, we isolated and characterized PSE-02, a novel dipeptide derived from PSE, and investigated its role as a potential key active compound. To evaluate its functional relevance in the context of the whole extract, we compared the effects of purified PSE-02 (analytical HPLC purity 95.1%; the remaining 4.9% consisted of low-abundance coextracted components that have not yet been structurally characterized and will be addressed in future work) with those of crude defatted PSE in an STZ-induced T2DM rat model. The administered dose of PSE-02 was equivalent to the estimated concentration of this compound in the HD PSE treatment group.

Both the low-dose LD and HD PSE-treated groups exhibited marked physiological improvements (Table 4). Compared with those in the DM group, rats in the low dosage PSE-treated group (LD) and high dosage PSE-treated group (HD) displayed reduced food and water intake, approaching the levels observed in the normal control (C) group. In terms of body weight, the final weights in the LD (379.9 ± 30.6 g) and HD (380.3 ± 19.3 g) groups were significantly higher than those in the DM group (376.7 ± 38.9 g), suggesting that PSE may attenuate diabetes-induced weight loss and metabolic catabolism.

**Table 4:** Effects of PSE at Low Dose (25 mg/kg Body Weight), High Dose (50 mg/kg Body Weight), and PSE-02 Group (0.06 mg/kg Body Weight) on Body Weight, Food Intake, and Water Consumption^c^

factors	CON	DM	PSE LD	PSE HD	PSE-02
initial weights, g	288 ± 5.57	286.88 ± 5.23	288 ± 4.9	290.25 ± 5.49	287 ± 5.48
final weights, g	402 ± 23.6^b^	346.71 ± 24.38^a^	379.86 ± 30.62	380.29 ± 19.29^b^	361.86 ± 45.35
food consumption, g	24.63 ± 2.14	28.13 ± 3.88	26.38 ± 3.29	25.88 ± 2.56	27.88 ± 2.96
water consumption, g	44.25 ± 8.22	60.54 ± 14.51	56.31 ± 9.55	54.70 ± 11.05	52.97 ± 6.88

a *p* < 0.05 versus control group.

b *p* < 0.05 versus DM group. Data are presented as mean ± SD (*n* = 8 per group).

c *CON: Normal control group. *DM: Diabetes mellitus group. *PSE LD: Low dosage PSE-treated group. *PSE HD: High dosage PSE-treated group. *PSE-02: PSE-02-treated group.

Biochemical analyses revealed significant improvements in the glycemic parameters (Figure 4). Compared with those in the DM group, FBG levels were reduced by 37.3% and 42.0% in the LD and HD groups (Figure 4A), respectively. As shown in Figure 4B, the HbA1c levels decreased by 14.2% and 26.3%, respectively, indicating a dose-dependent effect. In the PSE-02 group, FBG and HbA1c levels were reduced by 22.6% and 17.0%, respectively, supporting its role as a functional glucose-regulating molecule in PSE.^29^

**Figure 4:**
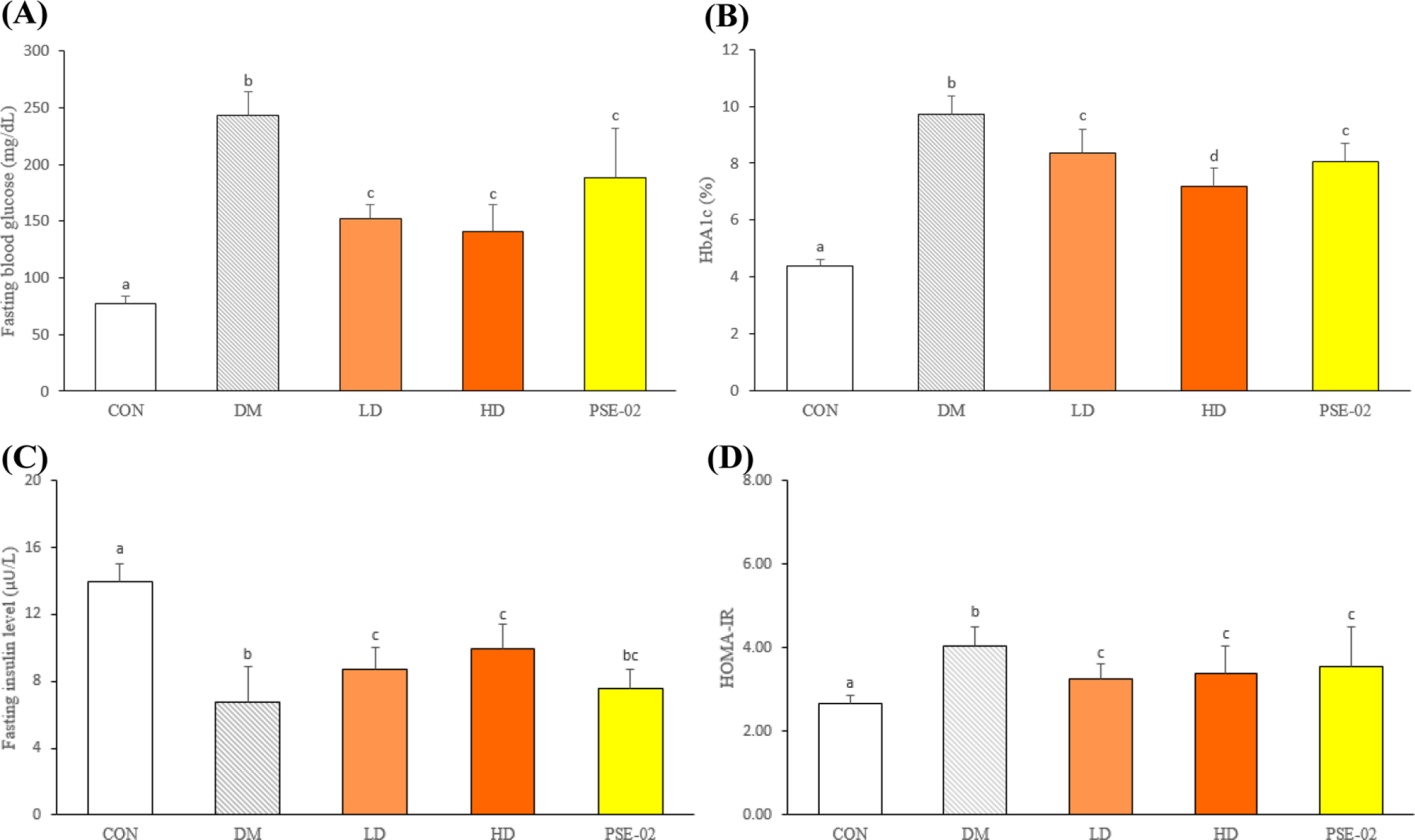
Effects of PSE treatment of diabetic rats on (A) FBG, (B) glycosylated hemoglobin, (C) fasting blood insulin level, and (D) HOMA-IR index at the eighth week of treatment. CON: Normal control group; DM: diabetes mellitus group, induced by STZ and HFD; LD: DM rats orally treated with PSE at a low dose (25 mg/kg); HD: DM rats orally treated with PSE at a high dose (50 mg/kg); and PSE-02: orally purified PSE-02 (0.06 mg/kg). Data are presented as mean ± SD (*n* = 8 per group). Different letters indicate statistically significant differences between groups (*p* < 0.05), as determined by one-way ANOVA followed by Tukey’s posthoc test. *CON: Normal control group. *DM: Diabetes mellitus group. *PSE LD: Low dosage PSE-treated group. *PSE HD: High dosage PSE-treated group. *PSE-02: PSE-02-treated group.

Regarding fasting insulin and HOMA-IR indices (Figure 4C,D), the LD and HD groups showed significant improvements, indicating enhanced insulin sensitivity and improved glucose homeostasis. Interestingly, the PSE-02 group did not show statistically significant differences in FBI and HOMA-IR compared with the DM group. This suggests that PSE-02 may have a limited role in modulating insulin secretion or resistance and that the effects observed in the PSE-treated groups may involve other bioactive constituents, such as antioxidant compounds, that contribute to improved insulin sensitivity.^30^

As shown in Figure 5, the OGTT further demonstrated the glucose-lowering potential of both PSE and PSE-02. At 60 min post-glucose challenge, blood glucose levels in the LD, HD, and PSE-02 groups were significantly lower than those in the DM group. The corresponding AUC values for glucose were reduced by 31.0%, 35.7%, and 28.5%, respectively, all reaching statistical significance compared to those in the DM group (*p* < 0.05). As shown in Figure 5B, the AUC for the glucose level was markedly reduced in both the LD and HD PSE-treated groups compared with that in the DM group, indicating enhanced glucose uptake and utilization.^29^ No significant differences were observed among the LD, HD, and PSE-02 groups, indicating that PSE-02 contributes substantially to the glucose-lowering effects of PSE under glucose overload conditions. However, the slightly greater improvements observed with crude PSE in some parameters (e.g., fasting insulin and HOMA-IR) suggest that other constituents in the extract may act additively or synergistically with PSE-02. Nevertheless, HOMA-IR and OGTT data provide only indirect estimates of insulin sensitivity in rodents. More direct dynamic assessments of insulin sensitivity, such as hyperinsulinemic-euglycemic clamp studies, were not performed in this work and represent important directions for future validation of the insulin-sensitizing effects of PSE and PSE-02. A limitation of the current design is the absence of an HFD-only, non-STZ group, which prevents a complete dissociation of diet-induced versus STZ-induced contributions to the diabetic phenotype; future studies incorporating this control will be important to further refine the mechanistic interpretation.

**Figure 5:**
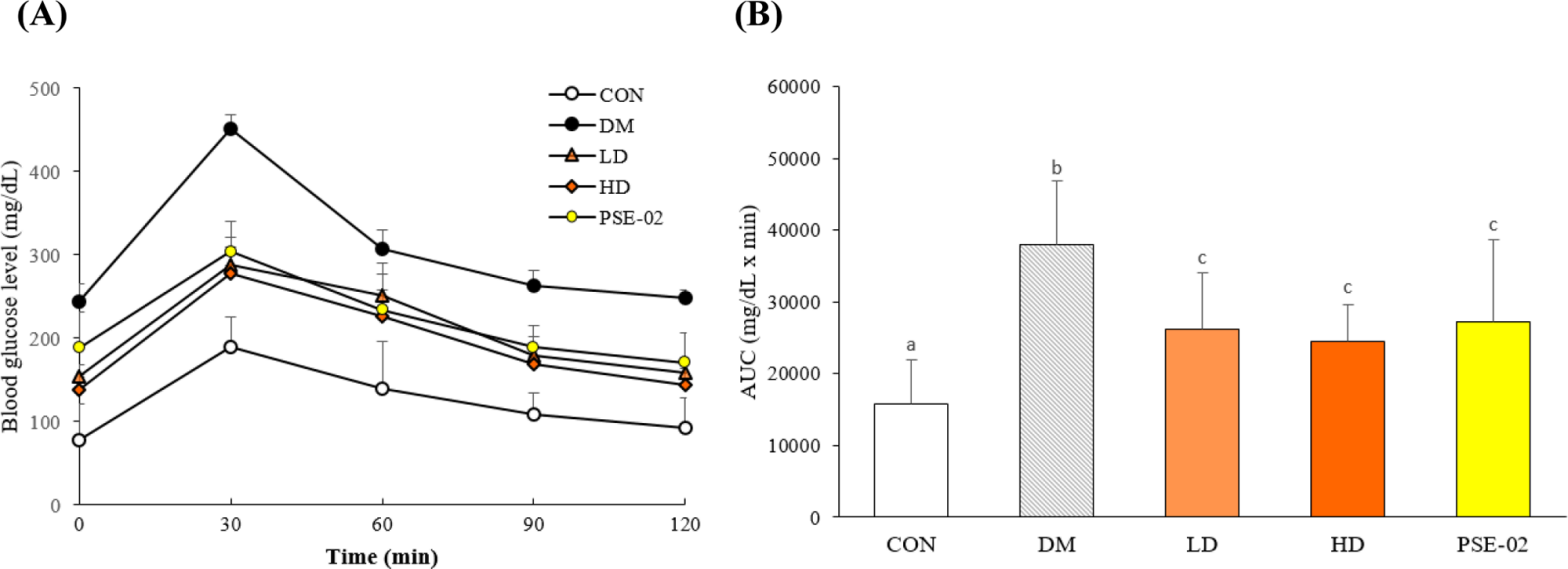
Effect of PSE on glucose metabolism in vivo. (A) OGTT curve. The blood glucose level was measured in tail vein blood at 0, 30, 60, 90, and 120 min. (B) AUC data were calculated for OGTT. CON: Normal control group; DM: diabetes mellitus group, induced by STZ and HFD; LD: DM rats orally treated with PSE at a low dose (25 mg/kg); HD: DM rats orally treated with PSE at a high dose (50 mg/kg); and PSE-02: orally purified PSE-02 (0.06 mg/kg). Data are presented as mean ± SD (*n* = 8 per group). Different letters indicate statistically significant differences between groups (*p* < 0.05), as determined by one-way ANOVA followed by Tukey’s posthoc test. *CON: Normal control group. *DM: Diabetes mellitus group. *PSE LD: Low dosage PSE-treated group. *PSE HD: High dosage PSE-treated group. *PSE-02: PSE-02-treated group. *AUC: Area under the curve.

### PSE-02 Enhances Insulin Signaling through the PI3K/PDK1/Akt Pathway in Skeletal Muscle

To elucidate the molecular basis of the observed hypoglycemic effects, Western blot analysis was performed on the skeletal muscle tissue to assess the phosphorylation status and expression levels of key proteins involved in insulin signaling, including IR, Akt, PI3K, PDK1, and GLUT4, which are the central regulators of insulin-stimulated glucose uptake.^31,32^

Figure 6A shows that the phosphorylation of the IR (p-IR/IR) was significantly elevated in the PSE-02 group, 1.86-fold higher than that in the DM group, and showed no statistical difference compared to that of the normal control group (*p* > 0.05), indicating substantial restoration of receptor activity. The LD and HD groups also exhibited marked increases with 1.46- and 1.79-fold enhancements, respectively, suggesting that crude PSE possesses basal insulin-sensitizing potential.

**Figure 6:**
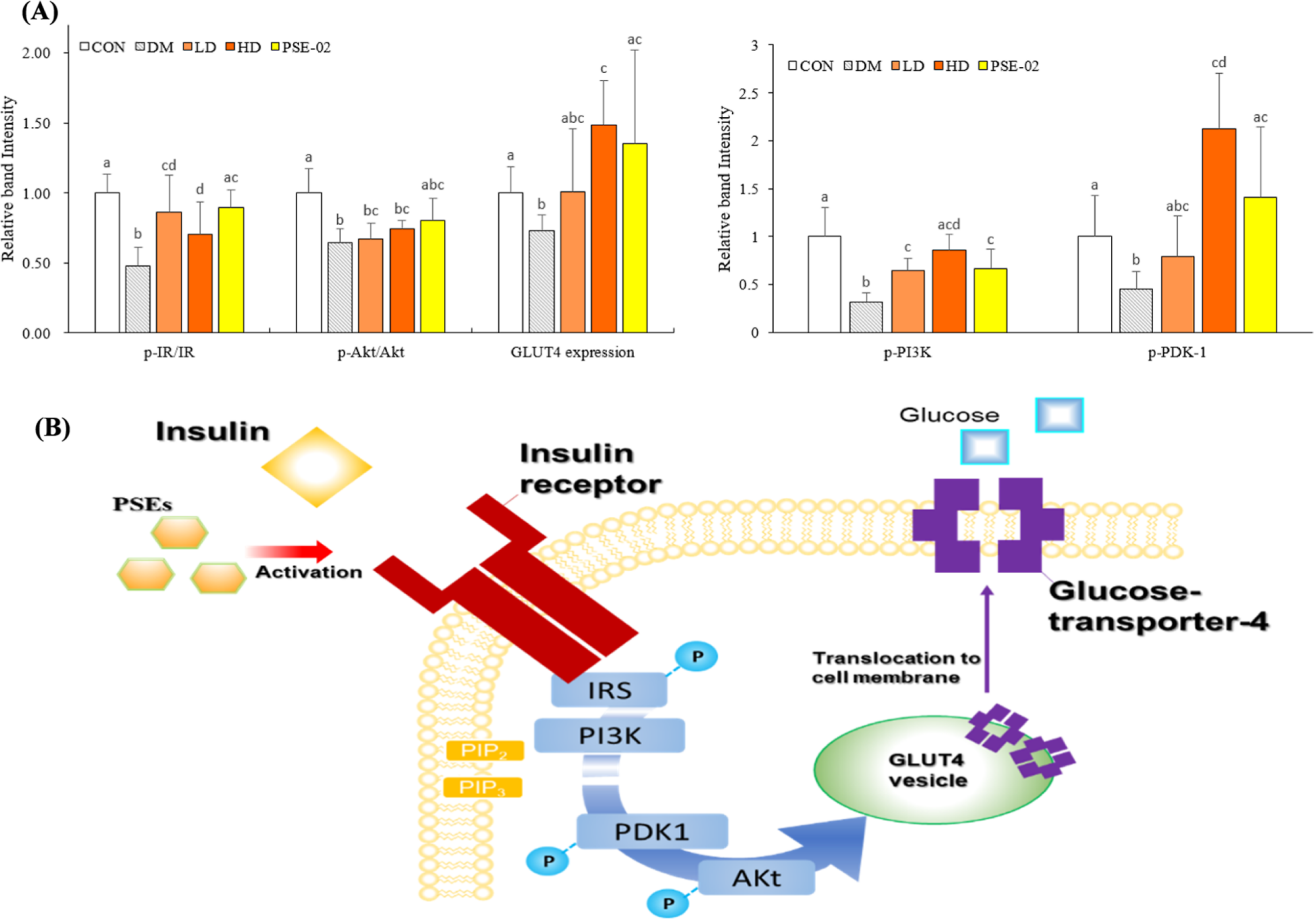
Effects of PSE on IR signaling and glucose clearance. (A) Western blot-based relative densitometric quantification of phosphorylated-to-total protein ratios (p-IR/IR, p-Akt/Akt, p-PI3K/PI3K, and p-PDK1/PDK1). Densitometric values were normalized to β-actin and are presented as mean ± SD (*n* = 8 per group); different letters indicate significant differences among groups (*p* < 0.05). (B) Proposed mechanism schematic showing that PSE promotes GLUT4 trafficking to the cell membrane via the IRS/PI3K/PDK1/Akt signaling pathway. *IRS: Insulin receptor substrate. *PI3Kα: Phosphoinositide 3-kinase. *PDK1: Pyruvate dehydrogenase kinase 1. *Akt: Protein kinase B. *GLUT4: Glucose transporter type 4.

For downstream signaling, the p-Akt/Akt ratio in the PSE-02 group reached 0.803, approaching that of the normal control, indicating a superior recovery of Akt kinase activity. The LD and HD groups exhibited increases of 1.014- and 1.15-fold, respectively, over the DM group, although these differences did not reach statistical significance. Nonetheless, the pattern was consistent with the RNA-seq data showing the regulation of *Pik3r1* and *Akt3*, supporting the alignment of transcriptional and translational responses.

GLUT4 protein expression was the highest in the HD group (2.03-fold vs DM, *p* < 0.05), followed by the PSE-02 (1.85-fold) and LD groups (1.38-fold), indicating that all treatments enhanced glucose transporter levels in skeletal muscle.^32^ The pronounced upregulation in the HD group may have resulted from amplified downstream signaling initiated by the PSE constituents.

Phosphorylation of upstream kinases PI3K and PDK1 was also significantly elevated (Figure 6A). The HD group showed the highest increase in P-PDK1 expression (4.69-fold over DM), while the PSE-02 group showed significant upregulation of both P-PI3K (2.09-fold) and P-PDK1 (3.11-fold), indicating enhanced activation of upstream insulin signaling components. Notably, both P-PI3K and P-PDK1 levels were higher in the HD group than in the PSE-02 group, suggesting that crude PSE may contain additional compounds that indirectly enhance these pathways, a hypothesis warranting further investigation.

In summary, both crude PSE and its purified derivative, PSE-02, restored insulin signaling in skeletal muscle by modulating multiple nodes within the PI3K/PDK1/Akt cascade (Figure 6B). The effects of PSE-02 on Akt phosphorylation and GLUT4 expression were consistent with the transcriptomic findings in C2C12 myotubes,^33^ particularly the regulation of *Pik3r1* and *Akt3*,^24^ supporting its role as a representative bioactive constituent with the potential for development as a functional ingredient in dietary interventions for diabetes management.

## Conclusions

This study identified PSE-02, a novel dipeptide isolated from defatted PSE, as an important bioactive constituent with antidiabetic potential. Both PSE and PSE-02 significantly improved glycemic control, insulin sensitivity, and glucose tolerance in STZ-induced T2DM rats. Mechanistically, PSE-02 restored insulin signaling in skeletal muscle through the activation of the PI3K/PDK1/Akt pathway, consistent with transcriptomic evidence showing the regulation of Pik3r1, Akt3, and mitochondrial-related genes. Although PSE-02 directly contributed to improved glucose uptake via GLUT4 expression, the crude PSE extract demonstrated additional upstream activation, suggesting the involvement of other synergistic compounds beyond PSE-02. Collectively, these findings support the use of defatted PSE as a functional food ingredient and highlight PSE-02 as a promising candidate for future development as a nutraceutical for diabetes management. However, these conclusions are derived from a rodent HFD/STZ model, and extrapolation to humans should be made with caution because of interspecies differences in gastrointestinal absorption, peptide stability against enzymatic degradation, immune and allergenic responses, and systemic exposure. Nevertheless, translation into practical applications will require comprehensive human clinical trials, systematic safety evaluation, and dose optimization to define human-relevant exposure ranges and establish a robust efficacy and safety profile for PSE and PSE-02 in the context of long-term dietary use.
